# Quantifying the Effects of Social Distancing on the Spread of COVID-19

**DOI:** 10.3390/ijerph18115566

**Published:** 2021-05-23

**Authors:** Talal Daghriri, Ozlem Ozmen

**Affiliations:** 1 Department of Industrial Engineering, Jazan University, Jazan 45142, Saudi Arabia; 2 Department of Industrial Engineering and Management Systems, University of Central Florida, Orlando, FL 162993, USA; Ozlem@ucf.edu

**Keywords:** COVID-19, disease spread, social distancing, agent-based modeling

## Abstract

This paper studies the interplay between social distancing and the spread of the COVID-19 disease—a global pandemic that has affected most of the world’s population. Our goals are to (1) to observe the correlation between the strictness of social distancing policies and the spread of disease and (2) to determine the optimal adoption level of social distancing policies. The earliest instances of the virus were found in China, and the virus has reached the United States with devastating consequences. Other countries severely affected by the pandemic are Brazil, Russia, the United Kingdom, Spain, India, Italy, and France. Although it is impossible to stop it, it is possible to slow down its spread to reduce its impact on the society and economy. Governments around the world have deployed various policies to reduce the virus spread in response to the pandemic. To assess the effectiveness of these policies, the system’s dynamics of the society needs to be analyzed, which is generally not possible with mathematical linear equations or Monte Carlo methods because human society is a complex adaptive system with continuous feedback loops. Because of the challenges with the other methods, we chose agent-based methods to conduct our study. Moreover, recent agent-based modeling studies for the COVID-19 pandemic show significant promise in assisting decision-makers in managing the crisis by applying policies such as social distancing, disease testing, contact tracing, home isolation, emergency hospitalization, and travel prevention to reduce infection rates. Based on modeling studies conducted in Imperial College, increasing levels of interventions could slow the spread of disease and infection. We ran the model with six different percentages of social distancing while keeping the other parameters constant. The results show that social distancing affects the spread of COVID-19 significantly, in turn decreasing the spread of disease and infection rates when implemented at higher levels. We also validated these results by using the behavior space tool with ten experiments with varying social distancing levels. We conclude that applying and increasing social distancing policy levels leads to a significant reduction in infection spread and the number of deaths. Both experiments show that infection rates are reduced drastically when social distancing intervention is implemented between 80% to 100%.

## 1. Introduction

In 2020, the novel coronavirus (COVID-19) has spread across the globe, taking lives and bringing devastating consequences. The pandemic is the most severe global health crisis in recent history since the 1918 influenza pandemic. During that pandemic, at least 50 million people died, and one-third of the world’s population was affected [1]. The extent of the coronavirus pandemic is yet to be seen as cases continue to rise exponentially. At the time of this study, there are at least three COVID-19 vaccines available. The distribution and administration to fully vaccinate the global population, however, is expected to take some time. Thus, using nonpharmacological strategies remains essential to reduce the spread of the disease and protect public health. Countries worldwide are implementing various social distancing policies, an important factor in reducing contact between people [2].

Data from China and Italy best show the effectiveness of various policies since they were the first countries to react to the pandemic. Italy was slower than China when implementing social distancing policies, and their delayed response resulted in a more severe disease spread. Lockdowns were gradually increased, with the final step occurring after thousands of people had contracted the virus and hundreds had died in Italy [3].

The pandemic had a devastating impact on the economy in many countries including the U.S. and China, leaving many unemployed. Quarter-on-quarter annualized economic growth rates predictions show −5% in Q1, −31.4% in Q2, 33.1% in Q3, and 4.1% in Q4 for the U.S. in 2020 [4]. The negative impact of social distancing policies on the economy is felt more severely by the business sectors that require close contacts, and the future looks bleak [5].

With such high economic costs and long-term results, strict social distancing policies are being questioned, where people have begun to wonder if they are worth it [6]. The data coming from China (as well as South Korea and Singapore) demonstrate the effectiveness of social distancing in slowing down the spread of the coronavirus; however, less strict interventions could have a less severe influence on the economy while still slowing down the spread [7,8,9].

Previous studies have analyzed these data to understand the trade-off between the benefits of social distancing and its negative impact on the economy in two scenarios [10,11]. In the first scenario, the social distancing measures are implemented for 3–4 months by isolating people with symptoms at home, quarantining people at home when they live in the same house as a suspected case, and social distancing the elderly and people most at risk. In the second scenario, no social distancing measures are implemented. The results from the first scenario (social distancing) show that 1.76 million fewer fatalities from COVID-19 would occur over six months than in the second scenario (no policy) [11]. Thus, our study aims to quantify the effects of different levels of social distancing on the disease spread based on the transmission and fatality rates of a specific location. To illustrate this, the numbers of cases are lower in some locations compared to other locations or countries, so flattening the curve could be achieved with different levels of social distancing policies. Governments need to apply only the required levels of social distancing to flatten the curve without applying more strict social distancing levels that would add no more significant difference and would also either impact the economy further or delay the reopening process. 

For this study, we developed an agent-based simulation model by using the NetLogo simulation platform to quantify the effect of social distancing on the spread of the COVID-19 disease. Our goals are to (1) to observe the correlation between the strictness of social distancing policies and the spread of disease and (2) to determine the optimal adoption level of social distancing policies. Simulation helps us conduct our study in silica, thereby inexpensively replicating the case study. Through these simulations, we can predict outcomes to provide recommendations for saving lives and improving healthcare services during the crisis. 

Our simulation model can be verified and validated with data from countries that have implemented social distancing and countries that cannot implement such guidelines due to any reason. This model can provide guidance in determining the course of actions in response to outbreaks and can support decision making by delivering outcomes effectively and efficiently. Our study seeks to quantify the safest level of social distancing that should be applied for better infectious disease control.

## 2. Materials and Methods

**Figure 1:**
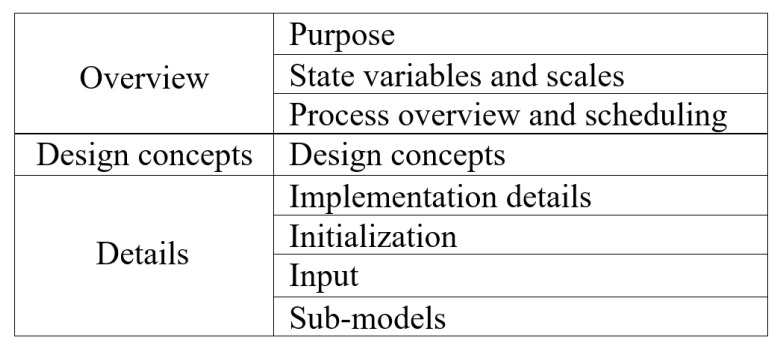
ODD Design protocol [12].

**Figure 2:**
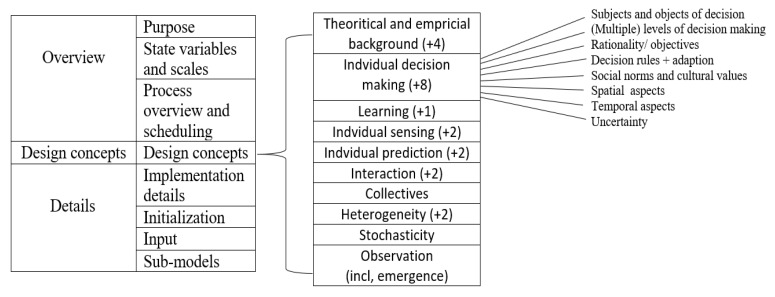
ODD+D protocol [12]. Plus sign (+) indicates numbers of added new questions (compared to the previous ODD).

We used agent-based modeling to capture the cause–effect relationship of COVID-19 and the well-known intervention of social distancing. This model is applicable for studying any location, as the model was designed to capture disease spread rates based on the input data of location of study. In addition, the model is different from other models because of its capability in quantifying the social distancing policies levels that are required to flatten the disease spread curve in the studied place. The model is also applicable for closed communities, such as districts, cities, and states, but it was not used in this study, as the model was used for the entire United States. Because of this, one of the future work goals is to be able to use this model for closed communities. Moreover, we used the extended version of the ODD (Overview, Design concepts and Details) protocol (Figure 1). ODD+D design protocol includes overview, design concepts, and detail plus decision Figure 2). In addition, ODD+D was extended to introduce the human decision-making behavior in reacting to the previous code. This protocol helps us study and analyze human behaviors regarding the decision-making process [12,13].

### 2.1. Overview

#### 2.1.1. Purpose

The purpose of this paper was to understand the effect of social distancing on the spread of COVID-19 by by using NetLogo agent-based modeling and determine the optimal level of social distacing policies that should be applied in the United States. Applying social distancing policies would mitigate the virus impacts by reducing the number of infected individuals. Implementing such policies requires knowledge that may be impossible to gain through simple mathematical linear equations or the Monte Carlo method because human society is a complex adaptive system with continuous feedback loops [14]. In such scenarios, an agent-based model are particularly helpful to investigate action–effect relationships.

#### 2.1.2. Entities, State Variables, and Scales

This model contains only a single agent—an individual. The individual has three states: healthy, infected, and recovered. Agents in the model are colored based on their status. The healthy agents are green, the infected are red, and the recovered agents are yellow. Each agent has one of two strategies: either they follow social distancing or they do not. Agents move randomly on the canvas. As described above, the agents must have a label representing their strategy: “S” represents agents who are following social distancing, while “N” represents agents who are not following the social distancing policy. The environment is a 2D grid of 32 × 16 cells. One cell can contain only one agent at a time. Agents can randomly move inside the model, pursuing the strategy they consider feasible for survival.

#### 2.1.3. Process Overview and Scheduling 

**Figure 3:**
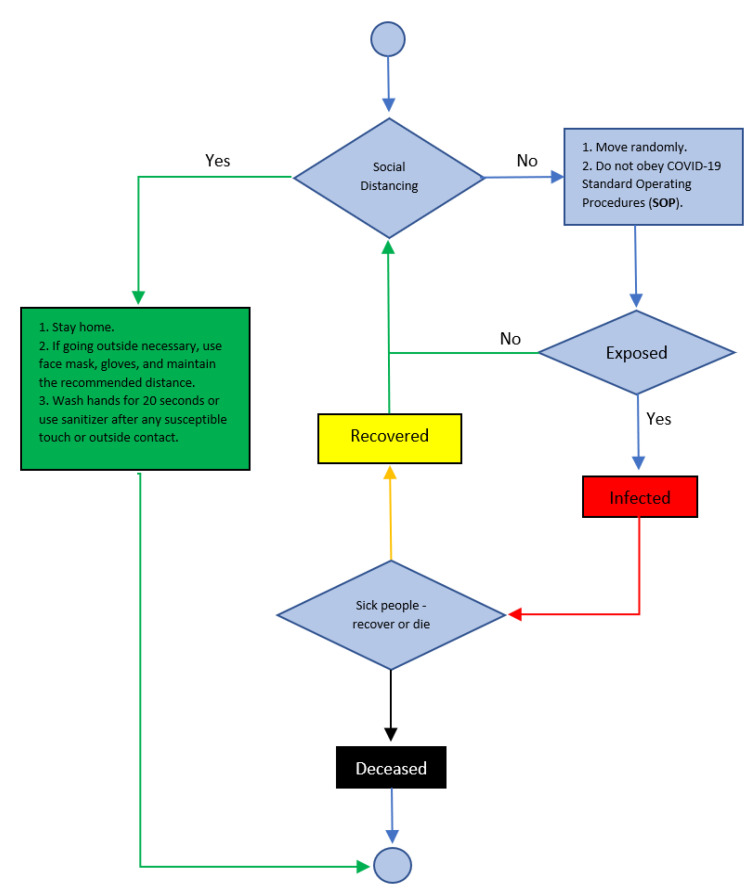
Process overview and scheduling.

Each agent must follow one of the two strategies (Figure 3): social distancing or no social distancing. A person moves randomly in the model and encounters another person. If one of the two people have COVID-19, the healthy person is affected and becomes sick. In the model, agents that follow social distancing guidelines refrain from interacting with others, while those who do not follow such guidelines interact with other agents.

### 2.2. Design Parameters 

This section describes the design concept of the model.

(1) Basic principle: The basic principle of the model is to understand the effect of social distancing on the COVID-19 spread. To understand this effect, we must use two types of strategies.
(2) Emergence: By employing the simple rule of social distancing, we can find the emergent structure of healthy and sick people in society.
(3) Adaption: People must adapt to the optimal strategy to remain healthy.
(4) Objectives: The objective for each person is to remain healthy and maintain their relationships.
(5) Learning: The agent learns how to survive by following social distancing.
(6) Prediction: Overall death and survival rates are predicted.
(7) Sensing: Agents can sense other agents who are infected with COVID-19.
(8) Interaction: Every agent can interact with other agents randomly.
(9) Stochasticity: The agent moves randomly in the canvas.
(10) Collectives: There are no collectives in the model.
(11) Observation: Infection, recovery, death, and survival rates are observed.

### 2.3. Details

#### 2.3.1. Initialization

Every experiment is initialized with different parameters (Table 1 and Table 2), and data from recent research were used for deriving some of the parameters. For instance, according to the Centers for Disease Control and Prevention (CDC), the total infections and fatality cases are 6,706,374 and 198,099, respectively, at the time of this study [1]. Since the fatality rate is about 3% was used in all experiments as a fixed value. In addition, Niklas Bobrovitz and his team at the University of Toronto assessed the disease test data to determine the proportion of infected people in the countries; it was found that the infection rate is 6% in the United States [15], and hence this figure was used as well for one of the parameters. 

#### 2.3.2. Input Data

Data were collected from the CDC website [1], as well as from a previous study [11].

#### 2.3.3. Submodels

The mathematical equations that are used to calculate the total recovered, infected population, and deaths after running the model are shown here. The total recovered population is calculated as follows:

(1) $$Trecovered=(\overset{total}{\underset{person=0\;\;}{\sum }}recovered=true)$$

(2) $$\;Trecovered\%=100(\overset{total}{\underset{person=0}{\sum }}\;\;recovered=true)/Pt$$

where T*recovered* is the total recovered population; “*recovered = true*” counts the cases if they are recovered; T*recovered*% is the percentage of total recovered from the population; and *Pt* is the total population. The total infected population is calculated as follows:

(3) $$Tinfected=(\overset{total}{\underset{person=0\;\;}{\sum }}infected=true)$$

(4) $$\;\;\;\;\;\;\;\;\;\;Tinfected\%=100(\overset{total}{\underset{person=0\;\;}{\sum }}infected=true)/Pt$$

where T*infected* is the total infected population; “*infected = true*” counts the cases if they are infected; T*infected*% is the percentage of total infected from the population; and *Pt* is the total population. The total deaths are calculated as follows:

**Total deaths** = Tinfected − T*recovered*

(5) $$Tdeaths=\left(\overset{total}{\underset{person=0\;\;}{\sum }}infected=true-\;\overset{total}{\underset{person=0}{\sum }}\;\;recovered=true\right)$$

(6) $$Tdeaths\%=100\left(\;\overset{total}{\underset{person=0\;\;}{\sum }}infected=true-\overset{total}{\underset{person=0}{\sum }}\;\;recovered=true\;\right)/Pt$$

## 3. Results

### 3.1. Social Distancing at 0%

**Figure 4:**
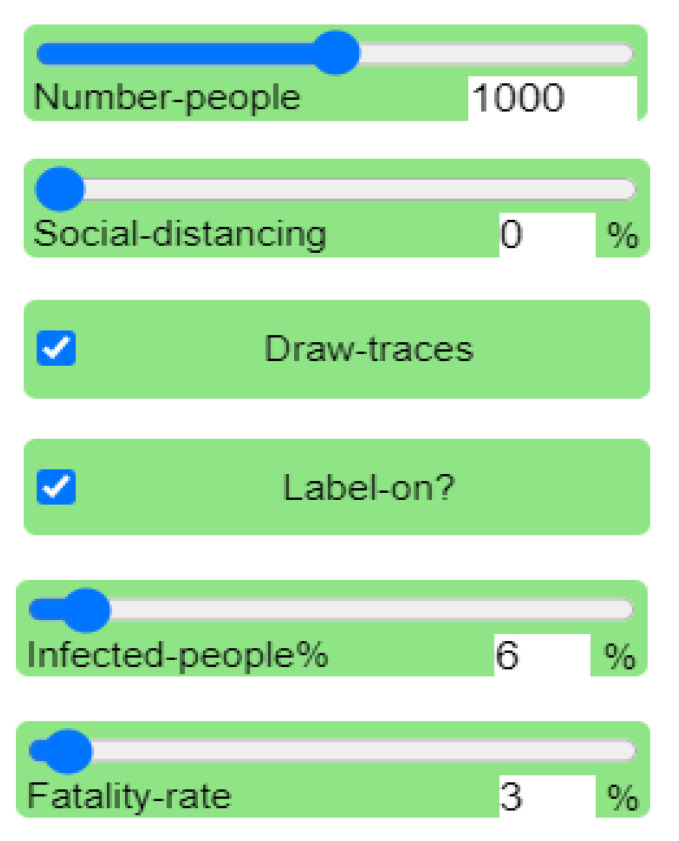
Sliders and switches of the NetLogo software interface.

**Figure 5:**
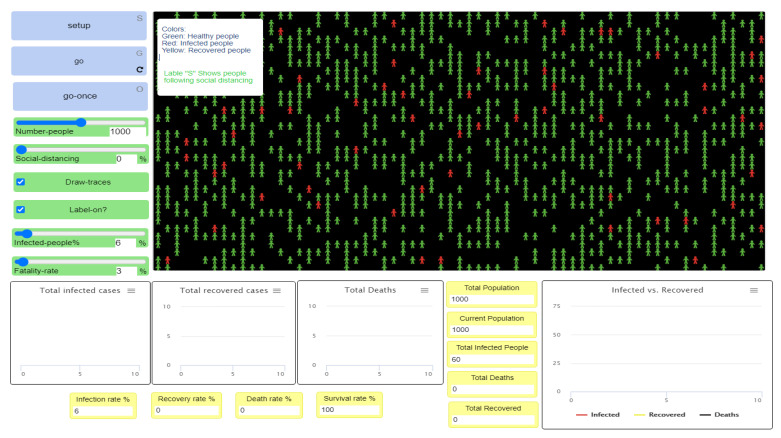
The Netlogo model interface [16].

The first experiment was run under a 0% level of social distancing (Figure 4). This means that no health intervention policies were used. The scenario presented disease transmission among the population without any preventive measures to stop or delay it. The model was run through the NetLogo website, as shown in Figure 5.

**Figure 6:**
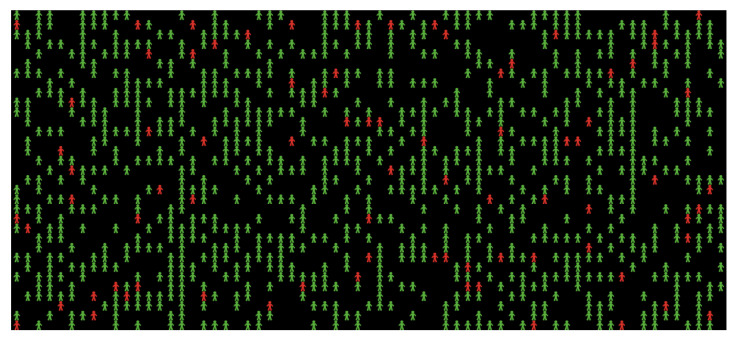
The initial visualization of the experiment.

In the first experiment, the social distancing percent slider was set to 0% to test the spread of the virus without following social distancing procedures, as shown in Figure 5. Since we are interested in studying and analyzing the effects of social distancing only, on the disease spread, the other parameters such as the number of people in the sample, the percentage of infected people, and the fatality rate remain fixed for all experiments. The initial population number is the same in all experiments (see Figure 6); however, the final population number differs in each experiment in terms of the number of infected individuals, recovered individuals, and deaths in the population. This can be observed visually in the simulation output. Figure 6 shows the initial visualization of the first experiment where red and green agents, respectively, represent the infected and healthy people. The disease transmission occurs through close contact between them; then, the infected cases either survive or die. Hence, the final visualization of the model renders a clear idea regarding the rates of healthy, infected, recovered, and deceased individuals at the end of the experiment.

**Figure 7:**
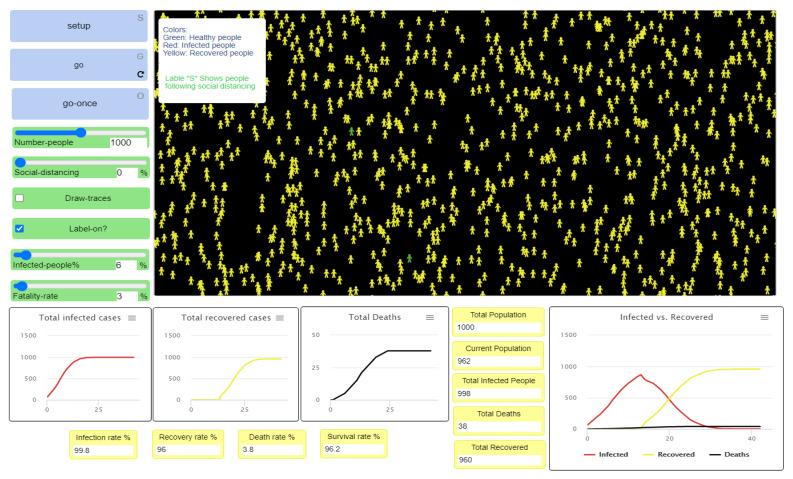
First experiment results of 0% social distancing.

**Figure 8:**
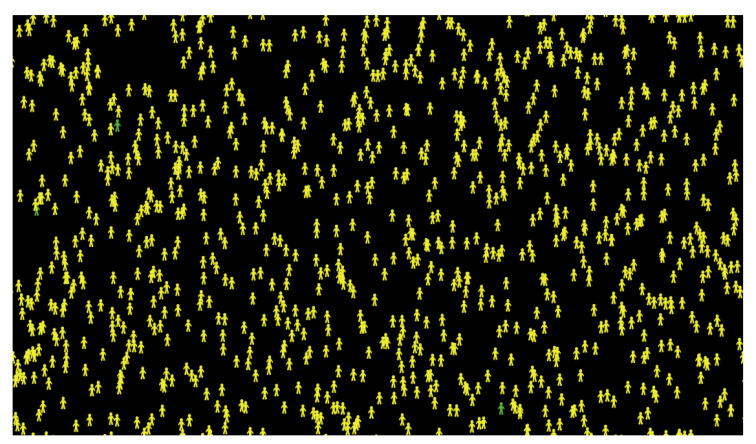
Final visualization of agents at 0% social distancing.

After completing the first experiment, as shown in Figure 7 and Figure 8, reading and analyzing the final visualization and statistical records became feasible. In other words, the number of infected people was too high because they did not socially distance themselves, even at only a 3% fatality rate. As we see, most of the infected cases recovered, which explains why most agents turned yellow at the end of the experiment while the number of healthy people who have never been infected remains small.

**Figure 9:**
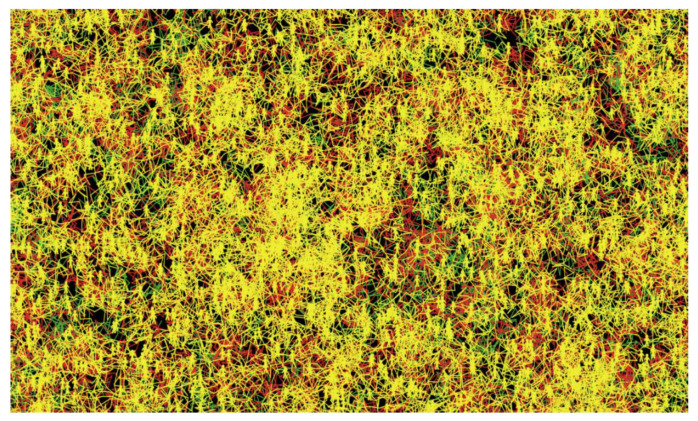
Movement patterns in the first experiment at 0% social distancing.

The movement patterns of the three different categories of agents were traced, as shown in Figure 9, where the red, yellow, and green movement patterns represent the motion of infected, recovered, and healthy agents, respectively. The infected and recovered people represent a considerable part of the final visualization, while the healthy people represent a small part of motion patterns. 

**Figure 10:**
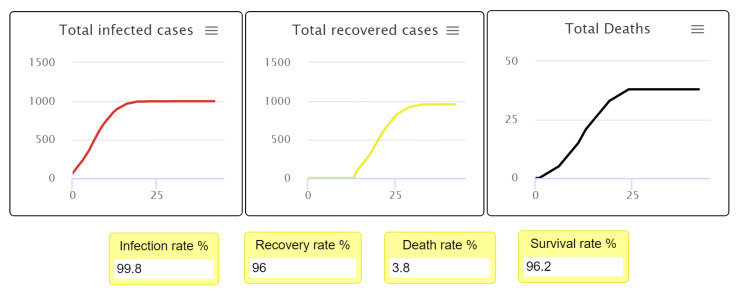
Infected, recovered, deceased, and survival population rate at 0% social distancing.

**Figure 11:**
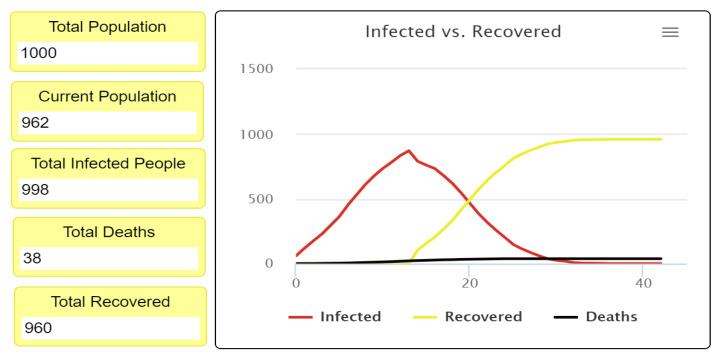
Infected versus the recovered people curve, when the fatality rate is 3% with 0% social distancing.

Figure 10 and Figure 11 show the statistical estimates and records of the total recovered, infected, and deceased populations. These records can be used to compare all experiment results and the efficiency of differing levels of social distancing. Understanding how the strictness of social distancing policy influences the transmission of disease also becomes possible, allowing decision-makers to create effective recommendations and regulations.

### 3.2. Social Distancing at 30%

**Figure 12:**
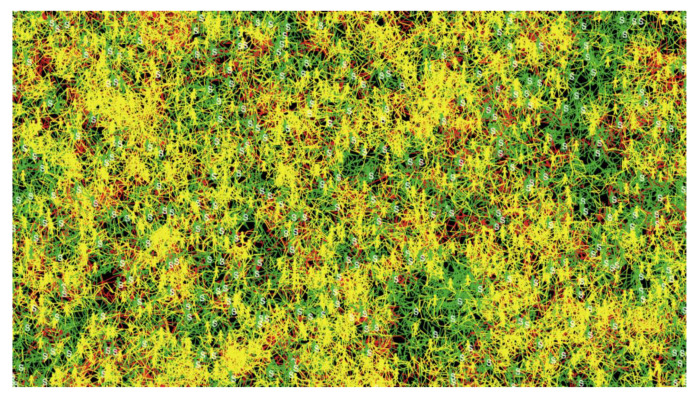
Movement patterns in the second experiment with 30% social distancing.

After the first results were received, the experiment was carried out again. This time, a higher level of social distancing was used, progressing from 0 to 30% without modifying any other parameters in the NetLogo model settings, as seen in Figure 12.

The final visualization of movement patterns in the second experiment (Figure 12) indicates that the infected and recovered agents’ movement patterns are lower than those in the first experiment. In contrast, the healthy agents’ movement patterns are more significant in the second experiment, meaning that a significant reduction of infection and an improvement of survival rate was achieved. 

**Figure 13:**
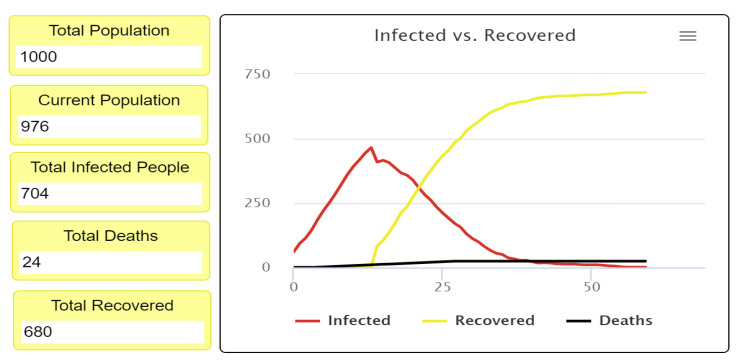
Infected versus the recovered people curve, when the fatality rate is 3% with 30% social distancing.

Based on Figure 13, using a 30% rate of social distancing reduces the number of infected people and deaths compared to the previous experiment. While the number of infected people was 995 in the first experiment, the number dropped to 701 infected cases in the second experiment. Therefore, the decline in infected numbers of people in the second experiment led to a decrease in deaths.

### 3.3. Social Distancing at 40%

**Figure 14:**
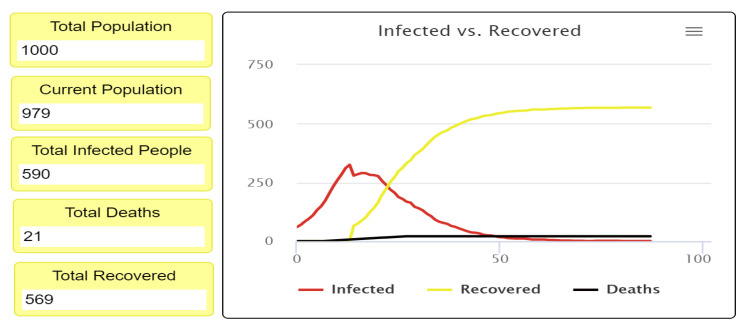
Infected versus the recovered people curve, when the fatality rate is 3% with 40% social distancing.

The next experiment increased the percentage of social distancing to 40%, resulting in further improvement and reducing the spread of disease to lower levels compared with the previous experiments, as shown in Figure 14. 

The previous three experiments prove that the more social distancing measures are applied, the fewer the number of cases, leading to a significant decrease in deaths. This is generally called flattening the curve.

**Figure 15:**
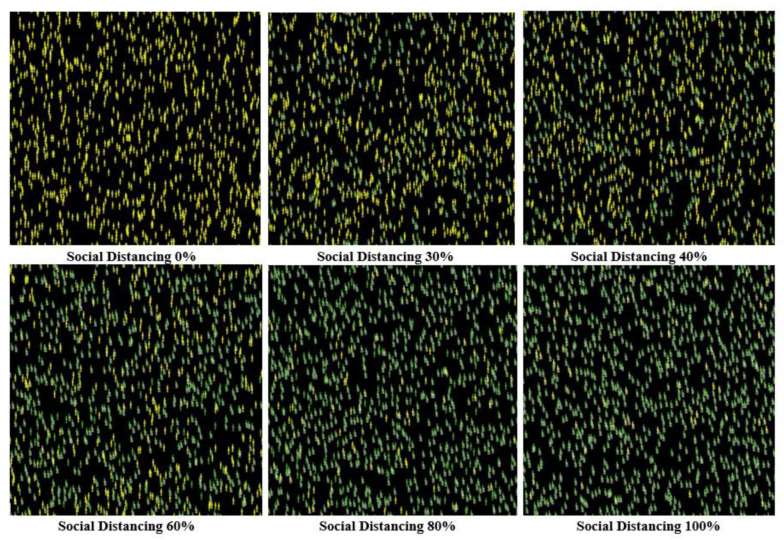
The final visual outputs of applying different levels of social distancing (without tracing individuals’ movement).

**Figure 16:**
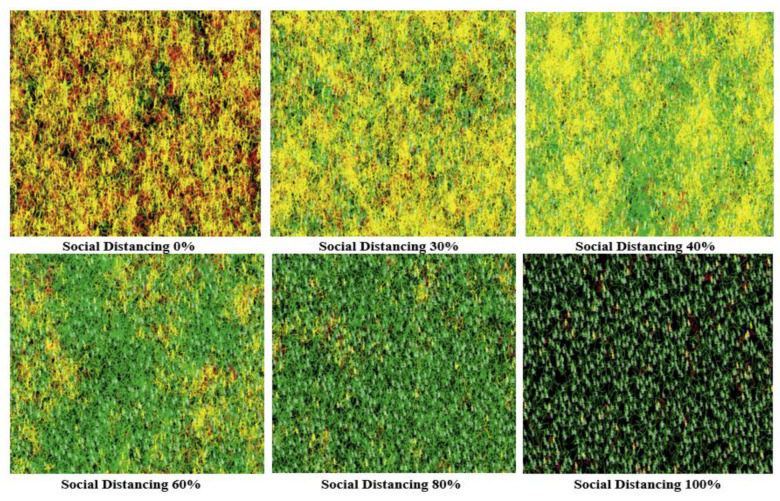
The final visual outputs of applying different levels of social distancing (with tracing individuals’ movement).

Here, the social distancing level was increased to 60%, 80%, and 100%, respectively, which led to reducing the spread of COVID-19 dramatically. The results show that applying 80% or higher of social distancing results in the lowest levels of infected cases and the highest survival rate and the lowest rate of death. These results suggest that the optimal level to reduce the disease spread to the lowest levels is 80% to 100% in the United States case. Figure 15 and Figure 16 show the different visualizations outputs of the six experiments, where the only difference between the two is that Figure 15 represents the agents without tracing their movement, while in Figure 16, the NetLogo model was set up to trace the agents’ movements.

**Figure 17:**
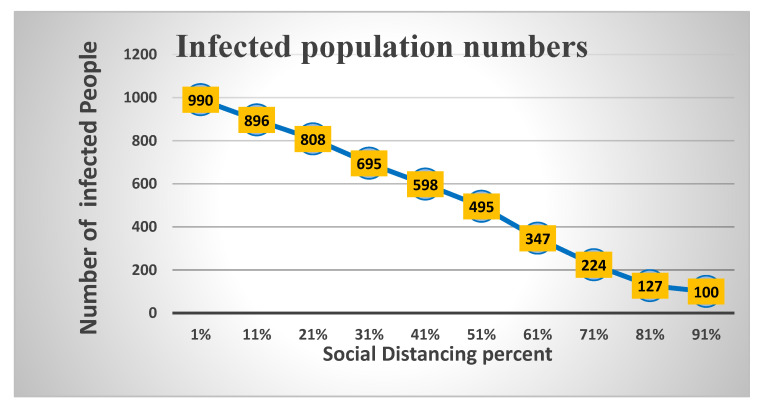
Effect of social distancing on infection rates.

**Figure 18:**
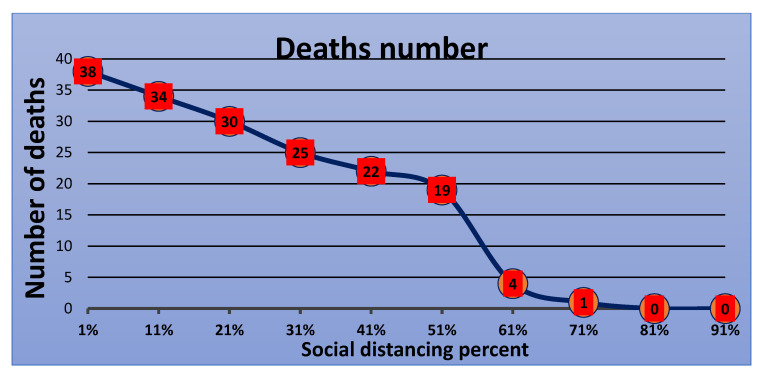
Number of deaths versus percentage of social distancing.

**Figure 19:**
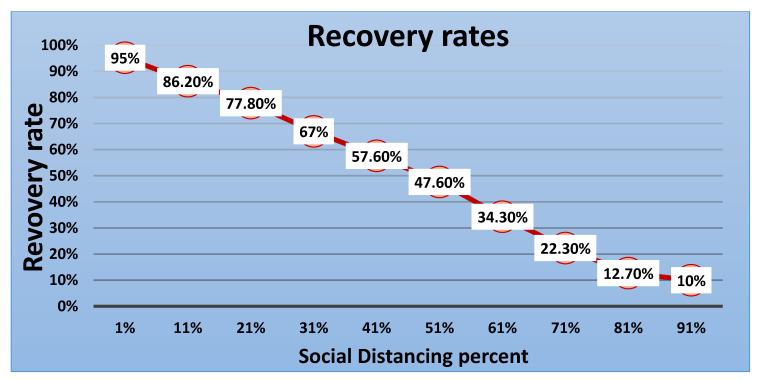
Recovery rate (recovered/total population) versus percentage of social distancing.

**Figure 20:**
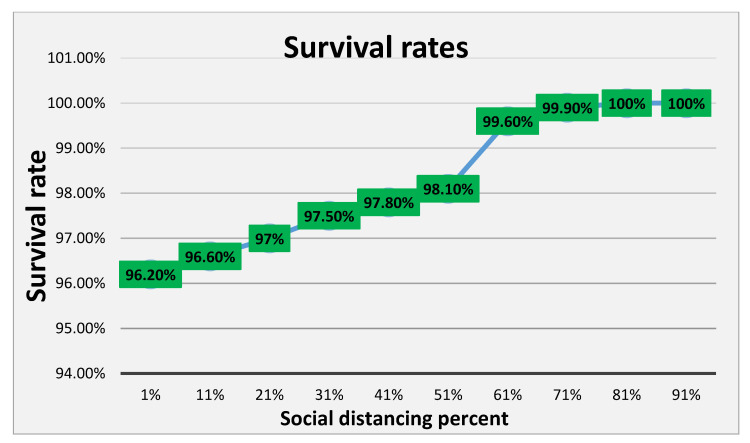
Survival rate versus percentage of social distancing.

Furthermore, additional experiments were conducted with ten varying levels of social distancing using the behavior space tool. The Figure 17, Figure 18, Figure 19 and Figure 20 show the results of these experiments. The infected cases curve in Figure 17 proves and confirms that increasing the social distancing percentage leads to a lower infection rate and decreases the deaths and recovery rates, as shown in Figure 18 and Figure 19, respectively. In comparing the previous six experiments, the results confirmed that the optimal level to flatten the curve is 80% to 100%. 

Figure 19 shows the rate at which people recovered after being infected by the virus and compares the different levels of social distancing policy; when social distancing was set at 91%, only 10% of the population became infected, resulting in a small number of people who needed to recover. Based on the declines of infected and deceased in Figure 20, the survival rate has dramatically improved (referred to as the current population). This evidence supports the governments and agencies’ choice to erect strict guidelines and procedures for social distancing.

## 4. Discussion

In this agent-based simulation model, we verified that social distancing can reduce the spread of COVID-19 and slow down the infection and fatality rates. We observed that implementing social distancing decreases infected cases. This helps in handling and treating severe patients [17]. In other words, a lower number of critical cases means that the hospital team would be able to handle all cases, resulting in an improved chance of survival for patients. In this way, the healthcare system is less likely to be stressed beyond the breaking point. 

We studied and simulated the social distancing behavior on discrete parameters ranging from 0% to 100%, i.e., from a case where no one is socially distanced to a case in which everyone socially distanced and followed recommendations by the public health agencies. These experiments clearly verified that social distancing at 0% implementation is very dangerous compared to the social distancing at 80% to 100% implementation for the United States case. At 0% social distancing, the spread of COVID-19 is much faster, and the fatality rate is much higher. These results allow us to conclude that we must enforce social distancing to reduce the spread of disease and assist in handling affected patients. Furthermore, countries or locations with lower infected and death rates may use lower strictness levels of social distancing policies to flatten the curve compared to the United States.

The proposed model was designed to observe the correlation between the strictness of social distancing policies and the disease spread in order to determine the optimal adoption level of social distancing policies. This model can be used to analyze any area, as it was designed to capture disease spread based on the input data of the study location. Furthermore, the model differs from others in its ability to measure the levels of social distancing policies needed to flatten the disease spread curve in the studied location. In addition, the model is applicable for the closed communities, such as states, cities, and districts. Since the model was developed through standard modeling tools (NetLogo software), it is flexible to be extended in the future, such as including another intervention strategy or modifying the limits of maximum number of agents. The model confirms and corroborates with the results of the widely studied epidemiology models such as the SEIR model [18,19]. In contrast to these traditional models, our model can be used to study heterogeneous populations to understand the effect of various policies on the spread of the disease and the number of deaths by overlaying different characteristics of the region and its population with different policies. 

One of the model’s most prominent features is that it can predict outcomes in silico and thus at a much lower cost than other studies involving participants and infrastructure in the real world. With this method, we can assist decision-makers and legislators with important factors by replicating a real-world situation, which is something impossible to do with linear mathematical equations that do not allow for heterogeneous agents and nonlinear interactions among them. Even though very few variables were considered in this agent-based simulation, the model’s speed efficiency remains satisfactory. This small sampling can help others acquire a useful, overall picture of the system. The study was conducted for the entire United States while ignoring other intervention strategies (e.g., wearing masks) to find out the pure effects of social distancing on the disease spread without any interaction with any other factors. Thus, the model could not quantify the effects of other interventions strategies, such as wearing masks, taking COVID-19 vaccines, or contact tracing.

## 5. Conclusions

In this model, we verified that social distancing affects the spread of COVID-19 by slowing down its spread and decreasing the number of active cases. According to our results, the optimal level of social distancing intervention should be at least 80% to reduce the infection spread and the number of deaths to the lowest numbers. Thus, high levels of social distancing effectively are required to reduce the spread of disease and flatten the curve in the United States. 

## 6. Future Work

We plan to extend the model to understand the effect of lockdowns on the economy, as lockdowns can result in severe issues with unemployment, survival, and growth rates. Our main purpose is to investigate how lockdowns affects the economy of such policies while keeping society safe.

## Figures and Tables

**Table 1:** The experiment factors and levels.

No.	Factor	Value
1	Total people	1000
2	Infection rate	6%
3	Fatality rate	3%
4	Social distancing strictness level	0%, 30%, 40%, 60%, 80%, 100%
5	Canvas	32 × 16 grid
6	Social distancing label	Displaced as label
7	Color	Yellow = Recovered 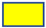 Red = Infected 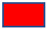 Green = Healthy 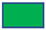

**Table 2:** The six experiments conditions/setting.

Experiment Number	Social Distancing Percent (Level)	Population	Transmission Rate	Fatality Rate
1	0%	1000	6%	3%
2	30%	1000	6%	3%
3	40%	1000	6%	3%
4	60%	1000	6%	3%
5	80%	1000	6%	3%
6	100%	1000	6%	3%

## Data Availability

Not applicable.
